# Extraction and purification of isochlorogenic acid C from Chrysanthemum morifolium using ionic liquid‐based ultrasound‐assisted extraction and aqueous two‐phase system

**DOI:** 10.1002/fsn3.768

**Published:** 2018-09-12

**Authors:** Yuqi Jiang, Zhunmei Ning, Shuang Li

**Affiliations:** ^1^ School of Perfume and Aroma Technology Shanghai Institute of Technology Shanghai China; ^2^ Shanghai Industrial Microbiology Institute Tech. Co., Ltd. Shanghai China

**Keywords:** aqueous two‐phase system, Chrysanthemum morifolium, isochlorogenic acid C, purification, ultrasonic‐assisted extraction

## Abstract

Ionic liquid‐based ultrasonic‐assisted extraction (IL‐UAE) was developed to extract and separate the isochlorogenic acid C (ICGA) from a cultivar of Chrysanthemum morifolium (*Chrysanthemummorifolium Ra Tnat*.). The influencing parameters, including IL concentration, liquid‐to‐solid ratio, and ultrasonic time, were optimized using response surface methodology. Of the ILs studied, 1‐butyl‐3‐methylimidazolium bromide [(Bmim)Br] exhibited the best extraction ability. The optimized conditions included liquid‐to‐solid ratio of 23.44:1, ultrasonic time of 48.99 min, and IL concentration of 0.65 mol/L. Under the optimal conditions, the extraction yield of ICGA could reach to 4.20 mg/g. An aqueous two‐phase system was applied for purification and separation of ICGA. The maximum extraction efficiency of 98.18% was obtained under the conditions of (NH_4_)_2_ SO_4_ of 4.5 g, pH of 3.0, and a temperature of 20°C at aqueous solution. Furthermore, the thermodynamic parameters showed that the purification of ICGA from salt‐rich phase to IL‐rich phase was a spontaneous and exothermic process. The results indicated that the proposed system is simple, rapid, and effective to serve as a viable and sustainable platform for the extraction and purification of ICGA from Chrysanthemum morifolium flowers.

## 1 INTRODUCTION

Chrysanthemum morifolium flowers (*Chrysanthemummorifolium Ra Tnat*.) are one of the commonly used herb plants and traditional medicines in China. It has been known for its medical values such as cold scattering, liver clamping, toxicity clearing, and eyes brightening (Delisle, Shipp, & Brodeur, 2015). As previously reported, Chrysanthemum morifolium has many antioxidant activities including resisting fatigue, improving the function of cardiovascular system, and lowering the levels of serum lipid (Fan et al., 2014; Wang & Xiao, 2013; Yu et al., 2013). Isochlorogenic acid C (ICGA), a di‐*O*‐caffeoyl derivative of chlorogenic acid, is a well‐known antioxidant that shows more potent bioactive effects than other isomers. Moreover, researchers have also found that ICGA and its isomers exhibit a broad‐spectrum antiviral potency against coxsackievirus (Zhou, Chen, Wu, Cao, & Zhang, 2016) and human immunodeficiency virus (Heyman et al., 2015; Robinson et al., 1996). In recent years, a large number of methods have been conducted on the extraction of caffeoylquinic acid, such as supercritical fluid extraction (Li, Yu, Yang, & Guo, 2012), ultrasonic‐assisted extraction (UAE) (Mazvimba, Ying, Cui, & Zhang, 2012), and pressurized liquid extraction (Wianowska, Typek, & Dawidowicz, 2015). However, there were very few researches aiming at extraction of ICGA. Meanwhile, traditional methods have suffered from many disadvantages such as high costs of instrumental consumption, operation, and maintenance. Moreover, the extraction and separation of these traditional methods need to be divided into two parts, which prolonged the reaction time.

Aqueous two‐phase system (ATPS), which is usually composed of polymer/polymer, polymer/salt, or salt/salt, has been recognized as an economical and efficient processing method (Atefi, Joshi, Mann, & Tavana, 2015). It provides a mild operating environment. Special structures like vesicles presented in an ATPS can also facilitate the separation process (Lu et al., 2015). Therefore, the utilization of ATPS is considered as a promising separation technique (Dong et al., 2015). In addition, ionic liquids (ILs) have emerged as alternatives for conventional organic solvents due to their characteristics such as negligible vapor pressure and volatility, low toxicity, adjustable polarity, general nonflammability, ease of recycling and manipulation, and high thermal and chemical stability (Lv, Jiang, Li, & Ren, 2012). Moreover, ILs can be designed by different combinations of cations, anions, and functional groups to allow for different purification processes. These designed ILs can be called task specific or functionalized ILs (Yang, Tan, Li, & Li, 2016). Thus, ILs have the properties of high extractability for both organic and inorganic compounds.

Ionic liquid‐based aqueous two‐phase system (IL‐ATPS), which is developed by Gutowski et al. (2003), is usually composed of hydrophilic ILs and inorganic salts, and IL‐ATPS has been widely applied as a more efficient and greener way for extraction and purification in one single procedure of various compounds, such as food additives and veterinary pesticides of agricultural products (Fan et al., 2014), pharmaceutical biomolecules (Tan, Li, Xu, & Xing, 2012), biochemical esterase (Lee, Khoiroh, Ling, & Show, 2017), heavy metal ions (Zheng, Tong, Wang, Zhang, & Yang, 2015), and protein (Tan et al., 2012).

**Figure 1:**
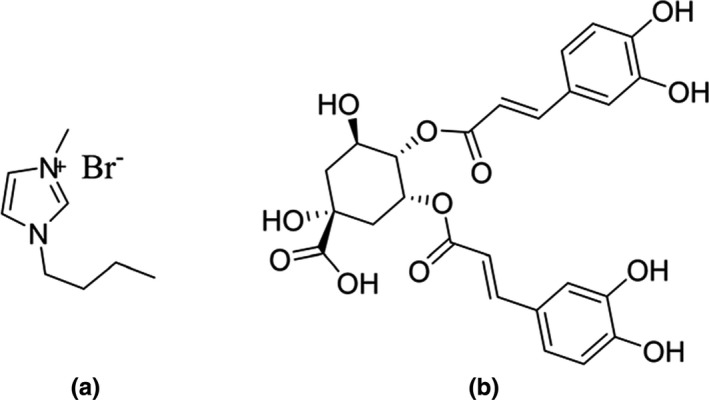
Chemical structures of (Bmim)Br 1‐butyl‐3‐methylimidazolium bromide (a) and IGCA (b)

**Figure 2:**
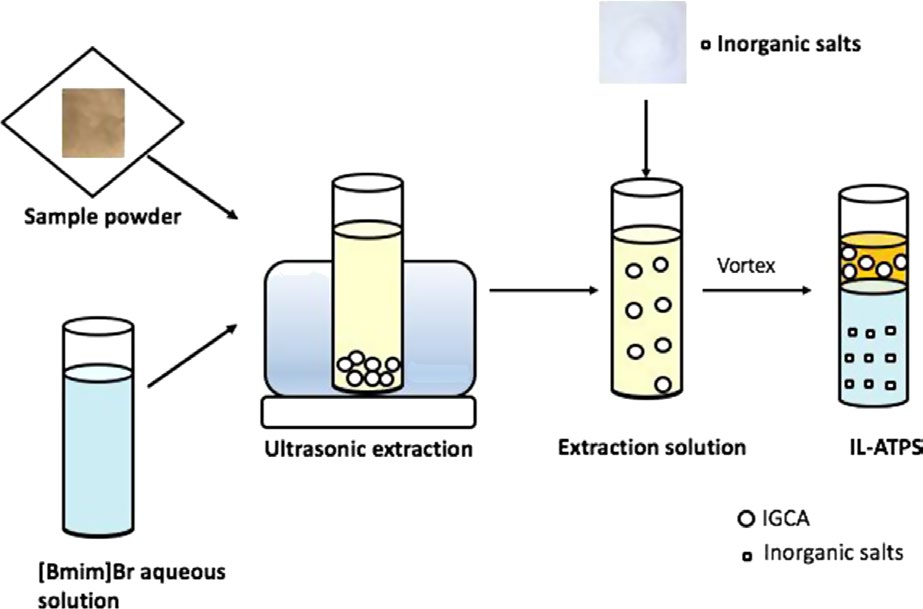
Schematic diagram depicting the route of ionic liquid‐based ultrasound‐assisted extraction (IL‐UAE) and ionic liquid‐based aqueous two‐phase system (IL‐ATPS)

In this work, an optimized single‐step procedure of IL‐UAE‐ATPS was studied to extract ICGA from a cultivar of Chrysanthemum morifolium. The studied IL was (Bmim)Br (1‐butyl‐3‐methylimidazolium bromide), the chemical structure of which is presented in Figure 1. The conditions (IL concentration, liquid‐to‐solid ratio, and ultrasonic time) were optimized using the response surface methodology (RSM). The key parameters including the salt type and amount, pH, and temperature were investigated in terms of their effect on the purification of ICGA. Moreover, the thermodynamics was explored simultaneously in the extraction procedure for the first time. The whole flowchart of extraction and purification of ICGA is shown in Figure 2.

## 2 MATERIALS AND METHODS

### 2.1 Reagents and materials

Chrysanthemum morifolium from Anhui Province was purchased from Shanghai Dedatang Sinopharm Co., Ltd. (Shanghai, China). The standard of ICGA (purity >98% by HPLC) was purchased from Chengdu Biopurify Phytochemicals Ltd. (Chengdu, China). (Bmim)Br (1‐butyl‐3‐methylimidazolium bromide) was purchased from Shanghai Cheng Jie Chemical Co., Ltd. (Shanghai, China). (NH_4_)_2_SO_4_, K_3_PO_4_, NaH_2_PO_4_, K_2_CO_3_, methanol, and ethanol were of analytical grade and were acquired from Sinopharm Chemical Reagent Co., Ltd. (Shanghai, China). Other reagents were all of analytical grade and used without further treatment. Deionized water was obtained with a Milli‐Q water purification system (Millipore) to prepare the sample solutions. The HPLC equipment used was Agilent 1260 HPLC system (Agilent Technologies, Germany).

### 2.2 Ionic liquid‐based ultrasonic‐assisted extraction

Dried flowers were pulverized into a homogeneous size by a grinder, sieved through a 60 mesh sieve, and dried at 45°C in the oven for 6 hr. Powders and IL solutions were mixed with a certain ratio in a centrifuge tube. The blend was placed in an ultrasonic bath at 25°C for some time to ensure the dissolution of the active ingredients. The extract solution was centrifuged at 12,000 g for 10 min, and the supernatant was filtrated through a 0.45‐μm microporous membrane and then injected into HPLC for analysis. The extraction yield of ICGA by ionic liquid‐based ultrasonic‐assisted extraction (IL‐UAE) was calculated according to Equation (1):

(1) Extractionyield=m/M

where the *m* is the mass (mg) of ICGA determined and *M* is the mass (g) of dried sample powder.

### 2.3 Ionic liquid‐based aqueous two‐phase system

Ten milliliters extraction solution and a given amount of salt were added to a 25‐ml centrifuge tube. The mixture was vortexed for 3 min to ensure the complete dissolution of the salt. Due to the incompatibility of IL solution and salt solution, and the low viscosity in the system, the phase separation can be achieved in a few seconds. The top IL‐rich phase was mainly composed of IL and ICGA occupying a small volume, and the bottom phase was salt‐phase solution containing the aqueous impurities occupying a large volume. The volume of each phase was noted down.

### 2.4 Analysis of ICGA

The two phases were separately withdrawn using pipettes for determining the concentration of ICGA by HPLC. The residues accumulated at the interface between two phases were discarded. All experiments were carried out at 25°C. The parameters are defined as follows.

The phase ratio (*R*), partition coefficient (*K*), and extraction efficiency (*E*) were evaluated. The parameters were defined as follows:

(2) $$R=V_{t}/V_{b}$$

(3) $$K=C_{t}/C_{b}$$

(4) $$E=\frac{K}{\left(K+1/R\right)}\times 100\%$$

where the *V*_*t*_ and *V*_*b*_ are the volumes of IL‐rich phase and salt‐rich phase, respectively, and *C*_*t*_ and *C*_*b*_ denote the measured ICGA concentration (mg/ml) in the IL‐rich phase and salt‐rich phase, respectively.

### 2.5 HPLC analysis

Chromatographic analyses were performed using an Agilent 1260 series HPLC system equipped with an Agilent Extend‐C_18_ column (4.6 mm × 250 mm, 5 μm). The injection volume was 5.0 μl, the temperature of the column oven was controlled at 30°C, and the flow rate of the mobile phase was maintained at 1 ml/min. The mobile phase consisted of 0.1% formic acid water (A) and 0.2% formic acid ACN (B) using a gradient elution of 10%–19% A at 0–16 min, 19%–20% A at 16–24 min, 10%–24% A at 24–48 min, 24%–50% A at 48–55 min, and 50%–10% A at 55–60 min. The UV wavelength was set at 384 nm. The data acquisition and analysis were performed by Agilent ChemStation software.

### 2.6 Comparison between IL‐UAE and other conventional extraction methods

In order to further compare the extraction yield of conventional heat reflux extraction (HRE) and UAE, the extractions of ICGA based on different solvents ([Bmim]Br aqueous solution, methanol, ethanol, and water) were performed. All the experiments were performed at the same conditions. Briefly, 0.42 g sample powder was mixed with 10 ml methanol, ethanol, or water ultrasonic treatment for 48.99 min. The extraction time for HRE was 2 hr at 80°C. The subsequent steps were the same as those in IL‐UAE.

## 3 RESULTS AND DISCUSSIONS

### 3.1 Single‐factor variation

Ionic liquid is usually composed of an organic cation in combination with an inorganic anion. The structure of IL has a significant impact on the extraction yield, and different structures may lead to a different extraction yield of analytes. According to Yang et al. (2013), the effect of changing the type of anion and the alkyl chain length of the cation of IL on the extraction yield was studied, and it was found that [Bmim]Br had the best extraction yield compared with other ILs. Therefore, [Bmim]Br was chosen for the subsequent study.

Structurally, the π‐bond of the heterocyclic structure in ILs is rich in numerous electrons, which can produce intermolecular binding through n–π or π–π stacking and promote the dissolution of effective substances in the samples (Chen, Cao, Gao, Qi, & Li, 2013). At the same time, ILs can help expansion and fragmentation of cell membranes in herbal plant cells in order to better dissolve active ingredients in the extractants (Albishri & El‐Hady, 2014). The concentration of [Bmim]Br is thought to be a crucial factor to affect the extraction yield of ICGA. [Bmim]Br with low concentration cannot extract completely due to their limited dissolving capacity. On the contrary, the excessive amount of [Bmim]Br is too viscous to penetrate through the solid sample powder and reduce the extraction yield of ICGA. Therefore, 0.75 mol/L was the suitable concentration for the further study.

**Figure 3:**
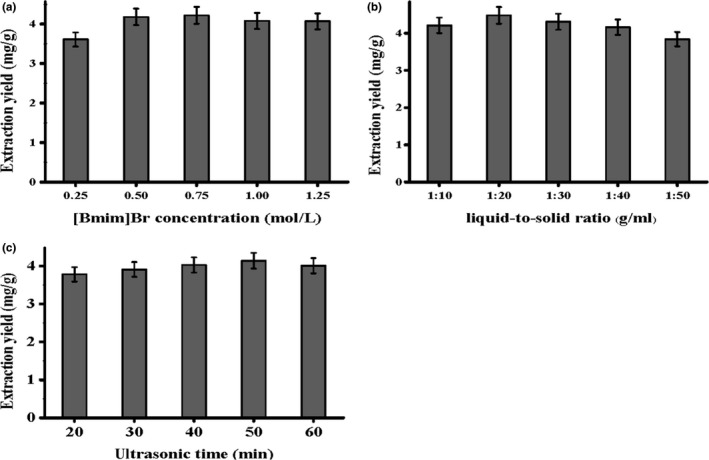
Single factor affecting the extraction yield of IGCA, including IL concentration (a), liquid‐to‐solid ratio (b), and ultrasonic time (c)

To some extent, the liquid‐to‐solid ratio and ultrasonic time play significant roles in promoting the extraction yield of the targeted compounds during the extraction. In general, a higher volume of solvent leads to a higher extraction yield. However, excessive solvent may dilute the extracts, which results in waste of solvents. Conversely, insufficient solvent may cause an incomplete extraction. As for ultrasound time, moderate sonication time is used to get maximum extraction yield. As shown in Figure 3, a liquid‐to‐solid ratio of 20:1 and ultrasonic time of 50 min were selected in the subsequent experiments.

### 3.2 Optimization of IL‐UAE conditions by RSM

To further study the interactions between the various factors, a Box–Behnken design approach and desirability function were used to optimize the IL concentration, liquid‐to‐solid ratio, and ultrasonic time in the IL‐UAE system. The Design‐Expert software was used. The experimental model defined the amount of different factors to be used to extract ICGA. The ranges for the extraction conditions were as follows: (A) liquid‐to‐solid ratio, 10:1–30:1; (B) ultrasonic time, 40–60 min; and (C) IL concentration, 0.25–0.75 mol/L. Table 1 shows the conditions of the 17 runs and the corresponding values of the test responses.

**Table 1:** Arrangement and results of the three‐factor/three‐level response surface design

Run	Factor A: solid‐to‐liquid ratio	Factor B: ultrasonic time (min)	Factor C: IL concentration (mol/L)	Extraction yield (mg/g)
1	0	0	0	4.04
2	−1	0	−1	2.99
3	0	0	0	4.02
4	−1	0	1	3.23
5	1	0	−1	3.05
6	−1	−1	0	3.02
7	0	1	1	4.04
8	0	1	−1	3
9	0	0	0	4.08
10	0	0	0	4.1
11	1	1	0	3.43
12	−1	1	0	3.53
13	0	0	0	4.09
14	0	−1	−1	2.99
15	0	−1	1	3.95
16	1	−1	0	3.83
17	1	0	1	3.87

As shown in Table 2, the model *F*‐value of 22.49 implied that the model was significant and there was only a 0.02% probability that a “model *F*‐value” of this large could occur due to statistical noise. Values greater than 0.1 indicated that the model terms were not significant and those less than 0.05 represented that the model terms were significant. Thus, the model explained the response adequately.

**Table 2:** Analysis of variance of Box–Behnken regression model

Source	Sum of squares	Degree of freedom	Mean square	*F*‐value	*p*‐value Prob > *F*	Remarks
Model	3.33	9	0.37	22.49	0.0002	Significant
A	0.25	1	0.25	15.1	0.006	
B	5.51 × 10^−3^	1	5.51 × 10^−3^	0.33	0.5809	
C	1.17	1	1.17	71.12	<0.0001	
AB	0.21	1	0.21	12.58	0.0094	
AC	0.084	1	0.084	5.11	0.0583	
BC	1.60 × 10^−3^	1	1.6 × 10^−3^	0.097	0.7643	
A^2^	0.71	1	0.71	43.38	0.0003	
B^2^	0.17	1	0.17	10.41	0.0145	
C^2^	0.57	1	0.57	34.89	0.0006	
Residual	0.12	7	0.016			
Lack of fit	0.11	3	0.037	31.21	0.0031	Significant
Pure error	4.72 × 10^−3^	4	1.18 × 10^−3^			
Cor total	3.45	16				

The magnitude of the absolute value of each coefficient in the equation reflected the degree of influence of each factor on the response value, and the positive and negative of the coefficient reflected the direction of influence. According to the significance of the regression coefficients of the quadratic polynomial model, IL concentration was found to be the most significant factor to affect the extraction yield of ICGA, followed by solid‐to‐liquid ratio and ultrasonic time. The quadratic terms (A^2^ and C^2^) were highly significant (*p* < 0.01), and B^2^ (*p* < 0.05) was also significant. Among the interaction terms, the interaction between AB (*p* < 0.01) was very significant with regard to the extraction yield of ICGA. The others were not significant (*p* > 0.05). The “lack‐of‐fit *F*‐value” of 31.21 implied that the “lack of fit” was significantly relative to the pure error and that there was a 0.31% chance that a “lack‐of‐fit *F*‐value” this large could occur due to statistical noise. It was clear that this model could be used to navigate the design space. The final predicted regression model between the extraction yield and these factors was given by the following Equation (5):

(5) $$\begin{array}{cc} Y= & 4.066+0.18A+0.03B+0.38C-0.23\text{AB}+0.15\text{AC}\\ & +0.02\text{BC}-0.41A^{2}-0.20B2-0.37C^{2} \end{array}$$

where *Y* was the extraction yield of ICGA and A, B, and C, respectively, corresponded to the coded values of the three independent variables solid‐to‐liquid ratio, ultrasonic time, and IL concentration.

**Figure 4:**
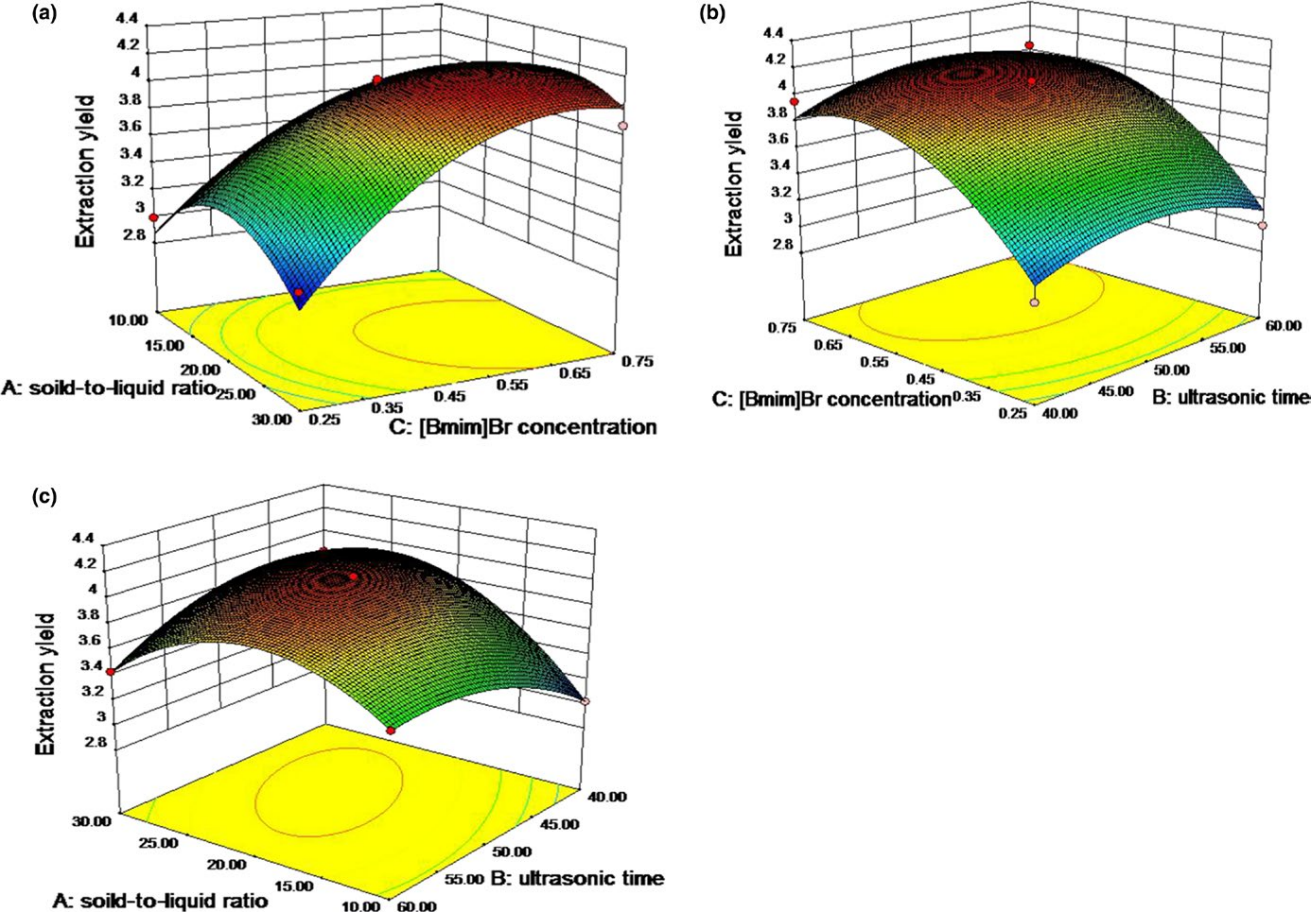
3D response surface plots showing the effects of variables on the average extraction yield of isochlorogenic acid C: (a) interaction between [Bmim]Br concentration (mol/L) and solid‐to‐liquid ratio (ml/g); (b) interaction between [Bmim]Br concentration (mol/L) and ultrasonic time (min); and (c) interaction between ultrasonic time (min) and solid‐to‐liquid ratio (ml/g)

The response surfaces for the effects of the independent variables on the average extraction yield of ICGA are shown in Figure 4. Based on the quadratic model, the optimal conditions for extraction of ICGA were as follows: liquid‐to‐solid ratio of 23.44:1, ultrasonic time of 48.99 min, and IL concentration of 0.65 mol/L. Under the optimal conditions, the extraction yield of ICGA can reach to 4.20 mg/g. This demonstrated that the model was adequate for predicting the expert optimization.

### 3.3 Comparison of IL‐UAE approach with conventional extraction methods

**Figure 5:**
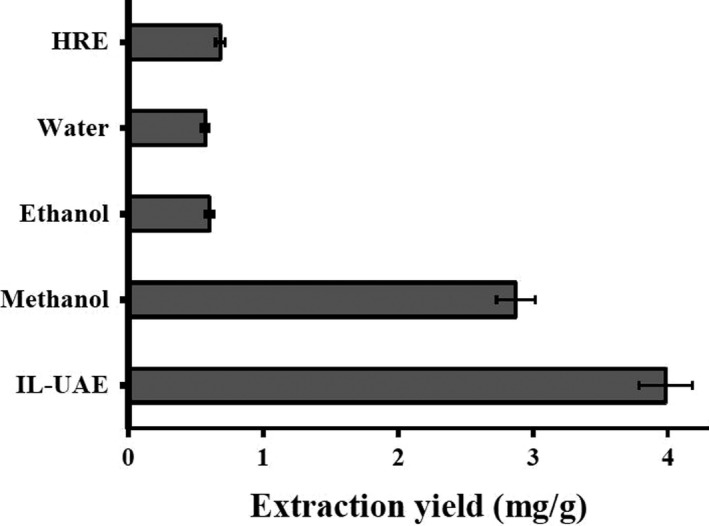
Comparison of different methods for the extraction of isochlorogenic acid C from white chrysanthemum

To further demonstrate the extraction yield of the IL (Bmim[Br]), a comparison was made between the proposed IL‐UAE and conventional UAE and HRE methods. The results are summarized in Figure 5. It can be seen that the method of IL‐UAE showed a higher extraction yield of ICGA compared with other conventional extraction methods, which can be explained that IL (Bmim[Br]) could offer great interaction with the ICGA. Moreover, as a nonvolatile and sustainable development solvent, IL (Bmim[Br]), which was used in the extraction of ICGA, is more environmentally friendly compared with conventional extraction solvents. Therefore, the proposed IL‐UAE method was chosen for the effective extraction of ICGA from Chrysanthemum morifolium.

### 3.4 Extraction and purification of ICGA by ATPS

Following the extraction of ICGA by IL‐UAE, it was further purified by ATPS. The type and amount of inorganic salt, temperature, and pH can be considered as the prominent factors influencing the purification efficiency of ICGA by ATPS.

### 3.5 Effect of salt type and amount

In order to choose an optimal inorganic salt for the enrichment of ICGA from Chrysanthemum morifolium, four different types of inorganic salt (Na_2_HPO_4_, (NH_4_)_2_SO_4_, NaHSO_4_, and Na_2_CO_3_) were considered. Among these salts, NaHSO_4_ and Na_2_HPO_4_ could not form ATPSs, and Bmim[Br]/(NH_4_)_2_SO_4_ and Bmim[Br]/Na_2_CO_3_ could observe the phenomenon of phase separation in just a few minutes. However, the Bmim[Br]/Na_2_CO_3_ system showed a significant change in color as the formation of phase separation. This may be due to the reason that there is a chemical reaction between alkaline Na_2_CO_3_ and acidic ICGA. Therefore, Bmim[Br]/(NH_4_)_2_SO_4_ system was selected for further studying the purification of ICGA.

**Figure 6:**
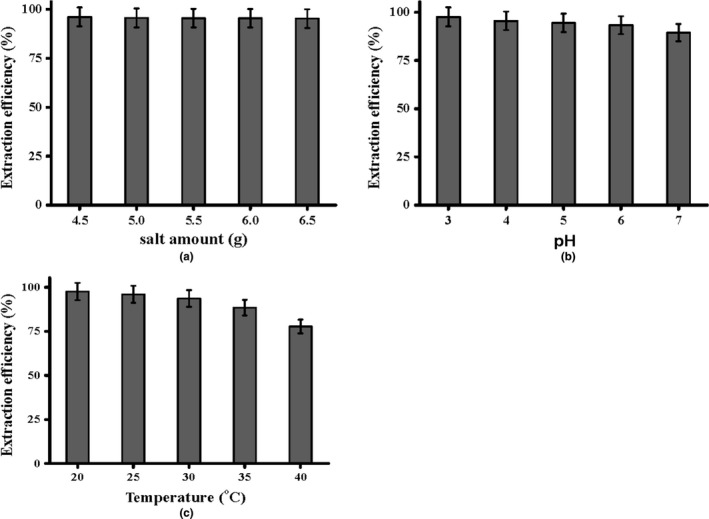
Effect of (a) salt amount; (b) pH; and (c) temperature on the extraction efficiency

To optimize the amount of (NH_4_)_2_SO_4_ added to form Bmim[Br]/(NH_4_)_2_SO_4_, the range of (NH_4_)_2_SO_4_ (from 4.5 to 6.5 g) was investigated. When the amount of (NH_4_)_2_SO_4_ was too low, ATPS could not be formed and the system was a homogeneous solution. With the amount of salt gradually increased, the solution was cloudy. Until the amount reached to 4.5 g, phase separation was formed. The upper phase was formed by the more hydrophobic IL solution, and the lower phase was consisted of the more hydrophilic salt solution. The amount of salt also directly affected the volume of the upper phase, thereby affecting the purification effect. As shown in Figure 6a, with the amount of (NH_4_)_2_SO_4_ increased, there was a slight decrease in the extraction efficiency of ICGA. This phenomenon may be due to the enhancement of salting‐out effect as the concentration increased in the bottom phase, and more free water molecules enter into the bottom phase, which leads to the increased viscosity and decreased contact area of the upper phase. Thus, the entry of target compound into the IL‐rich phase becomes more difficult, and the extraction efficiency decreased. Based on economic factors and extraction efficiency, 4.5 g of (NH_4_)_2_SO_4_ was selected to be the suitable amount.

### 3.6 Effect of pH

According to Tan et al. (2014), the pH of the aqueous solution was adjusted by the BR (Britton–Robinson) buffer that was mixed by 0.04 M phosphoric acid, 0.04 M acetic acid, 0.04 M boric acid, and 0.20 M sodium hydroxide. The extraction of ICGA was more stable in acidic conditions, so the range of pH 3.0–7.0 on the extraction efficiency was investigated. As illustrated in Figure 6b, the extraction efficiency decreased significantly. This may be due to the electrostatic interaction between ICGA and [Bmim]Br, and a change of pH can regulate surface electrical charge on ICGA, thereby affecting the distribution of the two phases. It is deduced that the electrostatic interaction between charged groups of ICGA and [Bmim]+ plays an important role in the partitioning performance of IL‐ATPS, which leads to the dependence of extraction efficiency on the change of pH. The maximum extraction efficiency could be achieved at pH of 3.0, which was selected for further purification of ICGA.

### 3.7 Effect of temperature

Temperature plays a crucial role in the mass transfer and solubility. A relative high temperature can enhance the mass transfer of target compounds and is also beneficial to the dispersion of IL. The results in Figure 6c indicated that the temperature had a significant effect on the extraction efficiency, and the extraction efficiency decreased with the temperature increased gradually. However, the effect of temperature on the phase volumes was not obvious. Thus, the changes of the extraction efficiency may be due to the different distribution performance of ICGA in the upper and bottom phases. When the temperature increased, the reduction in extraction efficiency may be explained by the degradation of ICGA. Finally, the extraction temperature of 20°C was selected for the further study.

**Figure 7:**
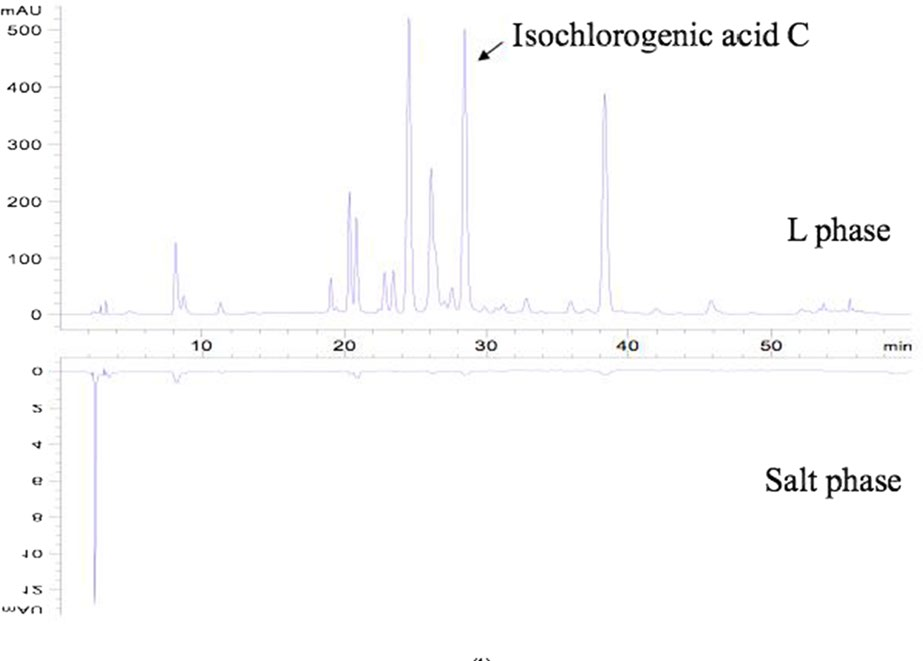
HPLC chromatograms for IL‐UAE‐ATPS sample IL phase and salt phase

A maximum extraction yield of 4.2065 mg/g and a extraction efficiency of 98.12% were obtained under the optimal conditions of IL‐UAE‐ATPS with the liquid‐to‐solid ratio of 23.44, ultrasonic time of 48.99 min, IL concentration of 0.65 mol/L, (NH_4_)_2_SO_4_ of 4.5 g, pH of 3.0, and extraction temperature of 20°C. By calculating the peak area of the HPLC chromatogram in Figure 7, ICGA was obviously enriched in top IL phase after the formation of ATPS, while it almost could not be detected in the lower phase. The retention time of ICGA was about 28 min, and the HPLC chromatogram exhibited that the ATPS did not interfere with the analysis of ICGA.

### 3.8 Thermodynamic parameters in extraction procedure

The parameters of thermodynamics during purification process were investigated to study the transfer process of ICGA from salt‐rich phase to IL phase. The Gibbs energy of transfer (∆*G*), from one phase to another, may be associated with its partition coefficients by Equation (6), where *R* is the universal gas constant and thermodynamic temperature (*T*) and distribution coefficient (*K*) were known.

(6) ΔG=−RTlnK

As shown in Equation (7), a linear correlation relation curve between ln *K* and 1/*T* was found within the temperature range of 20–40°C. The obtained equation was ln *K = *11,818 ×* *1/*T *− 33.401 with the correlation coefficient *R* ^2^ = 0.9927, where *−∆H*/*R* was slope and *∆S*/*R* was intercept. Over the investigated temperature range, the enthalpy change (*∆H*) was constant and the entropy change (*∆S*) also was calculated according to the intercept of relation curve. The detailed values of *T∆S*,* ∆G*, and *∆H* are shown in Table 3.

(7) $$\ln K=\frac{-\Delta H}{\mathrm{RT}}+\frac{\Delta S}{R}$$

**Table 3:** The thermodynamic parameters of isochlorogenic acid C transfer from the salt‐rich phase to the IL‐rich phase in aqueous two‐phase system

*T* (k)	*K*	Δ*G* (kJ/mol)	*T*Δ*S* (kJ/mol)	Δ*H* (kJ/mol)
293.15	800.27	−16.29	−81.96	−98.25
298.15	482.12	−15.31	−82.94	−98.25
303.15	296.00	−14.34	−83.91	−98.25
308.15	153.57	−12.89	−85.36	−98.25
313.15	69.88	−11.05	−87.20	−98.25

The thermodynamic parameters of the [Bmim]Br‐(NH_4_)_2_SO_4_ are listed in Table 3. All the *∆G* values were negative (*∆G *< 0), which indicated that the reaction of purification proceeded spontaneously from salt‐rich phase to IL‐rich phase.

Partitioning of ICGA in IL‐ATPS was marked by negative values for *∆H* and *TΔS*, in which the *∆H* could have greater value than the *T*Δ*S*. Hence, the purification of ICGA in IL‐ATPS from salt‐rich phase to IL‐rich phase was a spontaneous and exothermic process (Tan et al., 2014). The signatures of thermodynamics were agreement with the experimental results.

## 4 CONCLUSION

In this work, IL‐UAE coupled with the IL‐ATPS could be effectively applied in the extraction and purification of ICGA from Chrysanthemum morifolium. The optimized conditions of UAE were identified as a liquid‐to‐solid ratio of 23.44:1, ultrasonic time of 48.99 min, and IL (Bmim[Br]) concentration of 0.65 mol/L. Under the optimal conditions, the extraction yield of ICGA could reach to 4.20 mg/g. The maximum extraction efficiency reached 98.18% when 4.5 g (NH_4_)_2_SO_4_ was added to the system with the pH of 3.0 and the temperature of 20°C. Besides, the thermodynamics was explored simultaneously in the purification procedure for the first time, which could clearly explain the extraction mechanisms of IL/salt (Bmim[Br]/(NH_4_)_2_SO_4_) ATPS. Therefore, the IL‐UAE‐ATPS system is a sustainable, simple, and effective approach for the extraction and purification of ICGA from Chrysanthemum morifolium.

## CONFLICT OF INTEREST

The authors declare that they do not have any conflict of interest.

## ETHICAL REVIEW

This study does not involve any human or animal testing.
